# Physicochemical Properties and Oxidative Storage Stability of Milled Roselle (*Hibiscus sabdariffa* L.) Seeds

**DOI:** 10.3390/molecules23020385

**Published:** 2018-02-11

**Authors:** Nurul Hanisah Juhari, Mikael Agerlin Petersen

**Affiliations:** 1 Department of Food Science, Faculty of Science, University of Copenhagen, Rolighedsvej 26, Frederiksberg C, DK-1958, 1165 København, Denmark; map@food.ku.dk; 2 Department of Food Service and Management, Faculty of Food Science and Technology, University Putra Malaysia, Serdang 43400, Selangor, Malaysia

**Keywords:** Roselle seeds, storage stability, gas chromatography–mass spectrometry, volatile compounds, principal component analysis, physicochemical properties

## Abstract

Milled Roselle (*Hibiscus sabdariffa* L.) seeds of the UMKL cultivar were analyzed for proximate composition, water and oil absorption capacity, and the influence of storage conditions on storage stability. The storage stability was determined under four types of conditions: light/oxygen (air) (LO), light/nitrogen (LN), darkness/oxygen (air) (DO), and darkness/nitrogen (DN) while monitoring for seven consecutive months. During the storage period, the formation of volatiles was determined using dynamic headspace sampling and Gas Chromatography-Mass Spectrometry (GC-MS) analysis. In total, 85 volatiles were identified, mainly aldehydes, alcohols, ketones, furans, and acids indicating lipid oxidation. It is recommended that milled Roselle seeds should be flushed with nitrogen and stored in darkness. Under these conditions, the seeds can be stored for at least three months without changes in volatile profile. This is important to ensure the good quality of milled Roselle seeds for further commercialization.

## 1. Introduction

Roselle (*Hibiscus sabdariffa* L.) is an annual erect, bushy, herbaceous shrub that can be found in almost all warm countries such as India, Saudi Arabia, Malaysia, Indonesia, Thailand, Philippines, Vietnam, Sudan, Egypt, and Mexico. Roselle is a very useful plant [1,2,3]. The most exploited part of the Roselle plant is its calyces but an underutilized by-product, the seeds, could also be valuable. In Malaysia, calyces are normally processed to produce juices and various food products; the seeds are removed and disposed of as a by-product. The Roselle seeds are, however, also edible [4]. Previous studies have shown that Roselle seeds contain high levels of protein (13–35.4%), dietary fiber (18.3–42.6%), lipid (17.4–29.6%), and minerals (23.7–128 mg Ca, 596–672 mg P, 2.08–4.0 mg Zn, 0.21–3.1 mg Cu, 26.34–396 mg Mg, 0.08–0.18 mg Cr, and 0.36–0.51 mg riboflavin) [2,5,6,7,8]. In Nigeria, a decoction of the seeds is used to enhance lactation in cases of poor milk production and maternal mortality [9] and Nyam et al. [8] found that the seeds could be used as flour replacement for production of cookies enriched in protein and dietary fiber. In some countries, the seeds are roasted and consumed as a substitute for coffee [10].

Research done by Eltayeib and Elaziz [11] showed that Roselle seeds have a low content of free fatty acids, being indicative of low enzymatic hydrolysis, and are therefore well suited for preparation of food products.

The lipid in Roselle seeds has a high content of polyunsaturated fatty acids (70%) [12], which are highly vulnerable to oxidative deterioration. Before bringing production of Roselle seeds up to a commercial level, it is therefore crucial to know the stability of the product during storage. This can be done by identifying potential oxidation products that could contribute to the flavor i.e., as described by Holse et al. [13]. There are several factors that will influence the rate of lipid oxidation, namely fatty acid composition, moisture content, nature of the surface of the products, presence and activity of pro- and antioxidants, temperature, light, oxygen concentration and also relative humidity [13].

The overall aims of this study were to characterize the physicochemical properties of milled Roselle seeds and then to determine the influence of different storage conditions on the oxidative stability of milled Roselle seeds. From the storage experiment, suggested storage condition and shelf life will be given for the milled Roselle seeds.

## 2. Results and Discussion

### 2.1. Physicochemical Properties

The proximate composition, water absorption capacity (WAC) and oil absorption capacity (OAC) of milled Roselle seeds are presented in Table 1, together with values from literature. It is seen that dietary fiber is the major fraction in Roselle seeds, followed by protein, lipid, moisture, ash and carbohydrate. These values are grossly in accordance with other studies, although some differences are seen, probably due to different soil and climate conditions, agricultural practices [14], origin, and varieties. In general, Roselle seeds are a good source of dietary fiber compared to other common sources such as wheat and rice bran, oat and fiber from fruits [14]. Earlier studies have shown that Roselle seeds have high protein content compared with for example chickpea (*Cicer arietinum* L.), sunflower (*Helianthus annuus* L.), soybeans, groundnuts (*Arachis hypogaea* L.), and pigeon peas (*Cajanus cajan* L.) [15]. The lipid content of Roselle seeds is also considerable (16 to 30%).

The WAC and OAC are important since they will affect the texture, consistency, and mouthfeel when seeds are used as an ingredient in a food product, for example bread [6,7,16]. The levels of WAC in milled Roselle seeds are high compared to standard wheat flour measured in our lab (118%). A similar pattern is seen for OAC, where standard wheat flour has values of around 80%.

### 2.2. Monitoring of Oxygen and Nitrogen Levels in Packages

**Figure 1:**
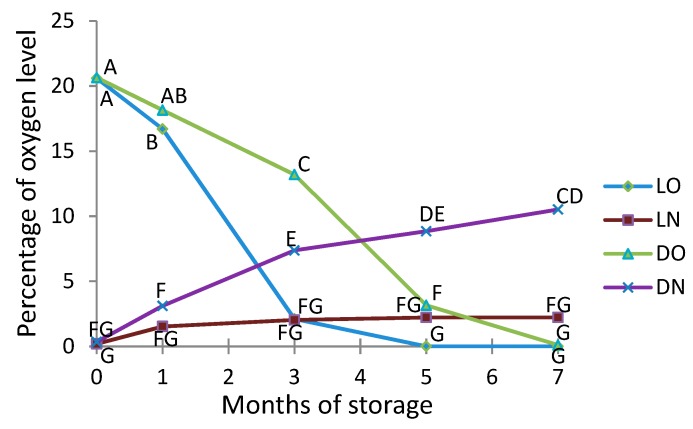
Effect of storage on oxygen levels in the headspace in milled Roselle seeds packed in the presence of oxygen (air) or nitrogen and stored under different light conditions (light or dark) for seven months. LO: samples stored in light in atmospheric air; LN: samples stored in light in nitrogen; DO: samples stored in darkness in atmospheric air; DN: samples stored in darkness in nitrogen. Observations marked with the same letter are not significantly different (*p* > 0.05).

The content of oxygen in the packages of milled Roselle seeds during storage is shown in Figure 1. For the packages that contained atmospheric air, a decrease in oxygen was seen during seven months of storage, most pronounced in samples stored in light. In the packages with nitrogen, the oxygen levels increased during storage, most in samples stored in the dark. The changes are a result of the balance between oxygen permeating through the packaging materials and oxygen removed due to oxidative processes within the package. The data in Figure 1 reveals that oxygen consumption is higher when packages are stored in light. In packages with air, the oxygen concentration decreases to a value close to zero, and it happens faster in packages stored in light. In packages with nitrogen, the higher oxygen increase in packages stored in the dark must be due to less oxygen consumption because the packages had identical geometry and therefore the same influx of oxygen. In fact, the oxygen level seems to stabilize in the samples stored in the light, indicating equal influx and consumption. In the samples stored in the dark, the oxidative processes are too slow to maintain a constant oxygen concentration.

### 2.3. Volatile Compounds 

**Figure 2:**
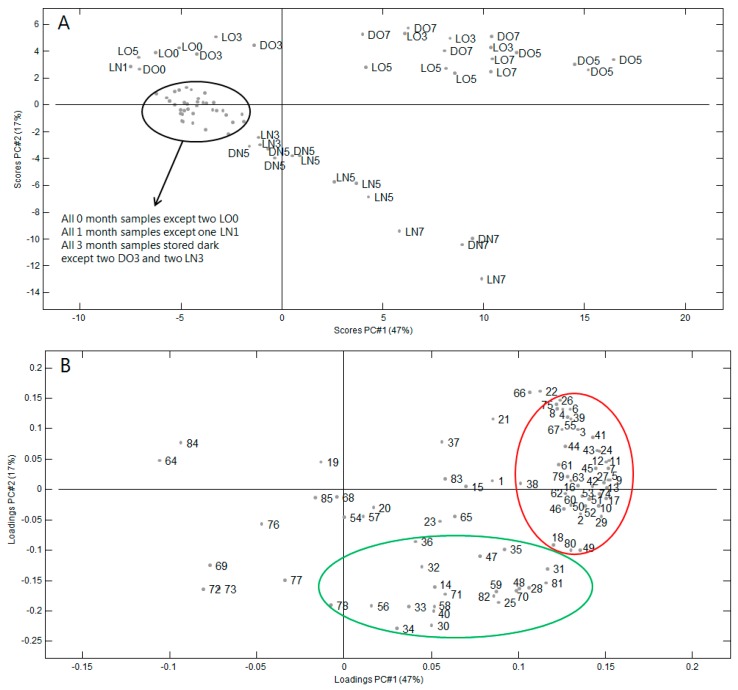
Principal component analysis (PCA) on autoscaled peak areas of volatiles in the headspace of milled Roselle seeds during storage. (**A**) score plot where LO: light/oxygen (atmospheric air); LN: light/nitrogen; DO: darkness/oxygen (atmospheric air); DN: darkness/nitrogen; numbers indicate storage time; (**B**) loadings plot where numbers refer to the compounds in Table 2.

A total of 85 volatile compounds, including aldehydes (18), alcohols (18), ketones (11), furans (8), acids (10), esters (6), terpenes (3), pyrazines (2), sulfur-containing compounds (2), lactones (4), and miscellaneous (3) were identified in the samples. Table 2 shows retention index, significance values and odor description of volatile compounds identified. Significant differences among samples were observed for almost all volatile compounds. These results are in accordance with our previous work on aroma profile of Roselle seeds [19] (Table 2, compounds in bold). A Principal Component Analysis was carried out using the Gas Chromatography-Mass Spectrometry (GC-MS) peak areas (Figure 2). The first principal component (PC1) explained 48% of the variance while PC2 explained 14% of the variance.

In the PCA score plot (Figure 2A), a clear tendency can be seen that samples move to the right when storage time increases (higher values of PC1). It is also seen that samples stored in nitrogen move to the lower right (increasingly negative values of PC2) while samples stored in air have rather constant (small) positive values of PC2. The influence of light is less clear from these plots. From the score plot, it can be concluded that storage of up to three months only has a small effect on the volatile profile, except if samples are stored in air and light (‘LO’).

When scores and loadings are compared (Figure 2A,B) it is evident that the levels of almost all compounds increase during storage. This is especially the case for the compounds in the red ellipse, while compounds in the green ellipse increase most in samples with nitrogen atmosphere. The compounds that generally increase (red ellipse) are mainly linear aldehydes (butanal, pentanal, hexanal, (*E*)-2-heptenal, octanal, (*E*)-2-octenal, nonanal, (*E*,*E*)-2,4-nonadienal, and (*E,E*)-2,-decadienal), Strecker aldehydes (2-methylpropanal, 2-methylbutanal, 3-methylbutanal, and 2-methylpentanal), furans (2-propylfuran, 2-butylfuran, 2-pentylfuran, and 2-hexylfuran).

**Figure 3:**
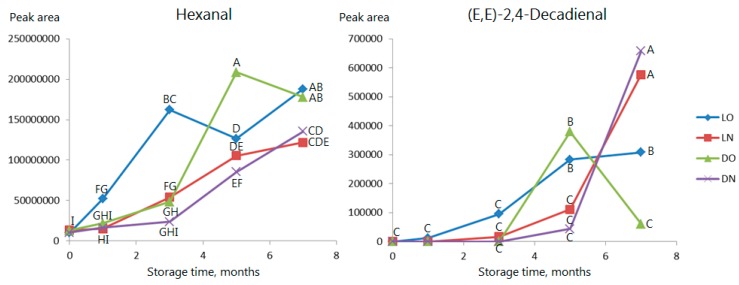
Effect of storage on levels of hexanal and (*E,E*)-2,4-decadienal in the headspace of milled Roselle seeds. LO: light/oxygen (atmospheric air); LN: light/nitrogen; DO: darkness/oxygen (atmospheric air); DN: darkness/nitrogen. Observations marked with the same letter are not significantly different (*p* > 0.05).

Up to three months, all linear aldehydes develop least in samples stored in the dark and most in the samples stored in air and light. After three months, the pattern becomes a little unclear, presumably because some aldehydes are broken further down, but, after seven months, all compounds except (*E,E*)-2,4-decadienal have the highest concentration in samples stored in air and light (‘LO’) (see Figure 3 showing hexanal and (*E,E*)-2,4-decadienal as examples). If samples are stored in nitrogen in the dark, no significant changes are seen during the first three months. Linear aldehydes are formed by oxidation of fatty acids. The oxidation can either be autoxidation, photooxidation or oxidation catalyzed by lipoxygenase, but since all create the same classes of secondary oxidation products, the present data cannot pinpoint which mechanism is the one dominating. It can be hypothesized that oxidation catalyzed by lipoxygenase is not very dominant, since this is the fastest. The fact that there is an effect of light indicates a combination of autoxidation and photooxidation to be the most prominent. Hexanal is often used as an indicator of oxidation, but many of the other aldehydes have also been reported to cause rancid off-flavors in a broad range of products [13,20,21].

**Figure 4:**
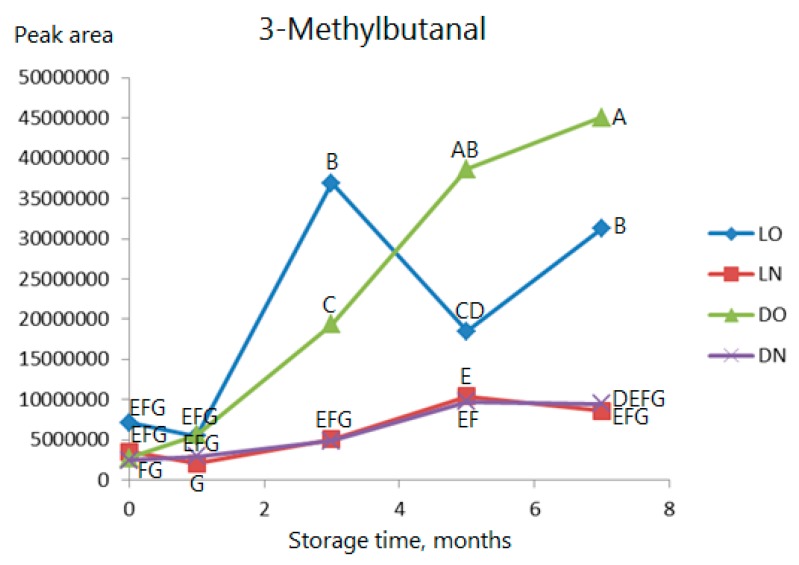
As Figure 3 but data for 3-methylbutanal. Observations marked with the same letter are not significantly different (*p* > 0.05).

Among the Strecker aldehydes, 2-methylbutanal, 3-methylbutanal, and 2-methylpentanal levels increase significantly in samples stored in air, while the increase is moderate or absent during storage in nitrogen. 2-Methylpropanal increases somewhat over time, but there is no clear relation to the storage conditions. As an example of a Strecker aldehyde, the development of 3-methylbutanal is shown in Figure 4. It is seen that, if stored in nitrogen, no significant increase will occur at any point of time. This is an even more pronounced effect of the nitrogen atmosphere than seen for the linear aldehydes. There is no clear effect of light. A positive effect of oxygen on the formation of Strecker aldehydes was also demonstrated by Wietstock et al. [22] during beer production and storage.

**Figure 5:**
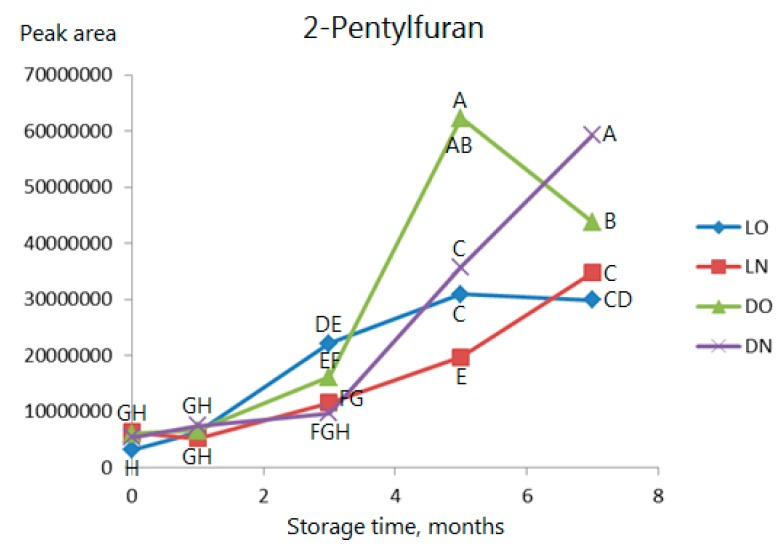
As Figure 3 but data for 2-pentylfuran. Observations marked with the same letter are not significantly different (*p* > 0.05).

The furans are generally increasing over time, and it is a common tendency that the levels are dropping in the last part of the storage when samples are stored with air (see Figure 5 showing 2-pentylfuran as an example). Again, there is no change during the first three months if the samples are stored in nitrogen. The effect of light is not clear.

The compounds that increase most in samples with nitrogen atmosphere (green ellipse in Figure 2B) are mainly alcohols (1-penten-3-ol, 2-heptanol, 2-octanol, 1-octen-3-ol, octanol, 2,3-butanediol, 2-decanol, benzyl alcohol, and phenylethyl alcohol) and acids (acetic acid, 2-methylpropanoic acid, and butanoic acid).

**Figure 6:**
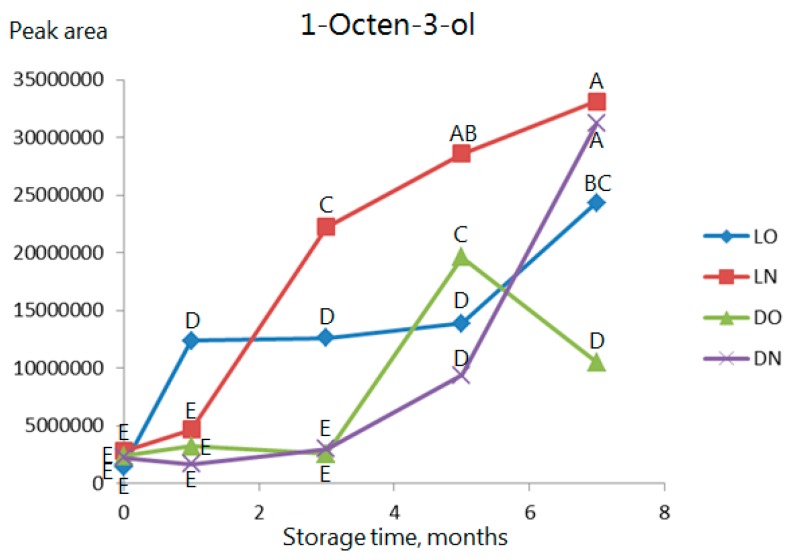
As Figure 3 but data for 1-octen-3-ol. Observations marked with the same letter are not significantly different (*p* > 0.05).

1-Octen-3-ol is shown in Figure 6 as an example of an alcohol. It is seen that, although it reaches the same high level after dark and light storage in nitrogen, the increase starts earlier in samples stored in light. Samples stored in dark exhibit no increase during the first three months.

**Figure 7:**
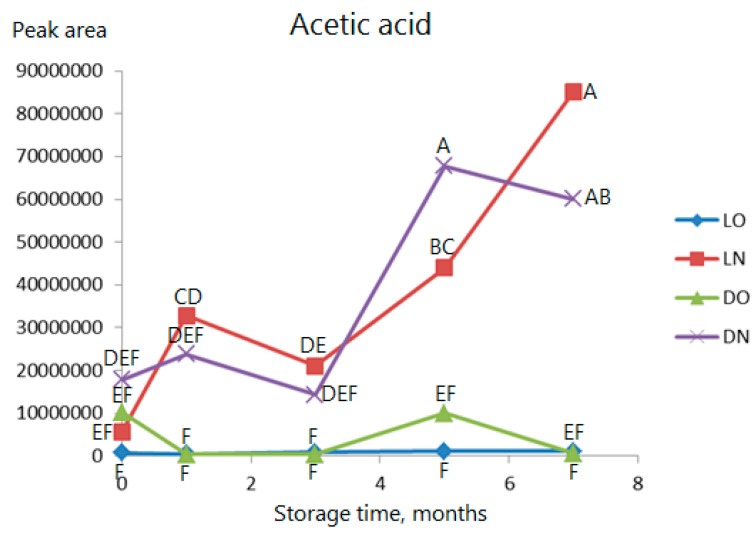
As Figure 3 but data for acetic acid. Observations marked with the same letter are not significantly different (*p* > 0.05).

Acetic acid is shown in Figure 7 as an example of an acid. When oxygen is present, there is almost no production of acids, but, in nitrogen, significant production is observed when stored more than three months.

Most of the mentioned alcohols and acids can be related to lipid oxidation and/or microbial growth. It could be hypothesized that microbial counts do not influence levels until late in the storage period, and the reason that it dominates in the packages with nitrogen could be that oxygen levels actually are highest late in the storage period (Figure 1). Microbiology was, however, not included in the present study, but could the subject of future studies.

Overall, the observations from the PCA are confirmed: There are practically no significant changes within the first three months of storage if samples are stored in nitrogen and in the dark. Furthermore, an effect of light was seen primarily on linear aldehydes and alcohols.

## 3. Materials and Methods

### 3.1. Chemical Standards

Chemical standards of volatile compounds, alkane standard mixture, petroleum ether, sodium hydroxide, ethanol, concentrated sulfuric acid, boric acid, hydrochloric acid, and acetone were obtained from Sigma-Aldrich/Merck (Merck KGaA, Darmstadt, Germany). Enzyme and celite, acid-washed, pre-ashed (Megazyme G-EL100 or G-CEL500) were supplied by Megazyme International Ireland Limited (Wicklow, Ireland).

### 3.2. Sample Materials

Sun dried Roselle (*Hibiscus sabdariffa* L.) seeds of the UMKL cultivar (obtained from HERBagus Sdn. Bhd., Penang, Malaysia) were chosen for the study. Identification of plant species was based on taxonomic descriptions and photographic illustrations by botanist Dr Shamsul Khamis from Institute of Bioscience, University Putra Malaysia. Roselle seeds were received from HERBagus as one batch of 6 kg from which representative samples were drawn. The samples were manually sorted to remove dirt and other extraneous matter. The samples were stored at −24 °C in glass jars flushed with nitrogen. Prior to the physicochemical analysis and storage experiment, the seeds were taken out from the freezer and milled for 90 s using a laboratory blender Model 38BL41 (Waring, CT, USA). The particle size of the milled Roselle seeds was as follows: 2.2% of the milled seeds had a particle size less than 75 µm; 13.3% between 75 µm and 160 µm; 13.6% between 160 µm and 250 µm; 27.5% between 250 µm and 500 µm; 39.5% between 500 µm and 1000 µm, and only 3.9% had a particle size bigger than 1000 µm.

### 3.3. Chemical and Physical Analyses

#### 3.3.1. Proximate Analysis

Proximate analysis of the milled Roselle seeds was performed according to the standard Association of Official Analytical Chemist (AOAC) method [24]. Moisture content (hot-air oven method), ash (dry ashing method), lipid (Soxhlet extraction), protein (Micro-Kjeldahl method), total dietary fiber [25] and carbohydrates (by difference) were analyzed and calculated. All measurements were conducted in triplicate. The results were expressed as a percentage (wet weight).

#### 3.3.2. Water and Oil Absorption Capacities

Bhat and Yahya’s method [16] was employed for the determination of water and oil absorption capacity. One gram of the milled Roselle seeds was mixed with distilled water or oil (10 mL) in a centrifuge tube and allowed to stand at room temperature (25 °C) for 1 h. After this time period, samples were centrifuged (200× *g* rpm for 30 min). WAC or OAC were expressed as percentage of water or oil absorbed by 1 g of the milled seeds. All measurements were carried out in triplicates.

### 3.4. Packaging and Storage Conditions

The milled Roselle seeds were packed in transparent plastic laminate bags (PA/PE 20/70) with an oxygen transmission rate (OTR) of 32 mL/m^2^/24 h/atm (23 °C, 75% RH) and a water vapor transmission rate (WVTR) of 1.0g/m^2^/24 h (23 °C, 85% RH). Each bag was filled with 85 g of milled Roselle seeds and then either sealed under atmospheric conditions (21% oxygen) or flushed with nitrogen (<1% oxygen). The bags were packed using a Komet Digi-Gas Packaging Machine (Mirovac X200, Plochingen, Germany). Two replicate bags were prepared for each storage condition and for each month of analysis. The samples were represented as follows:

LO (Light/Oxygen): samples stored in light in atmospheric air,
LN (Light/Nitrogen): samples stored in light in nitrogen,
DO (Darkness/Oxygen): samples stored in darkness in atmospheric air,
DN (Darkness/Nitrogen): samples stored in darkness in nitrogen.

**Figure 8:**
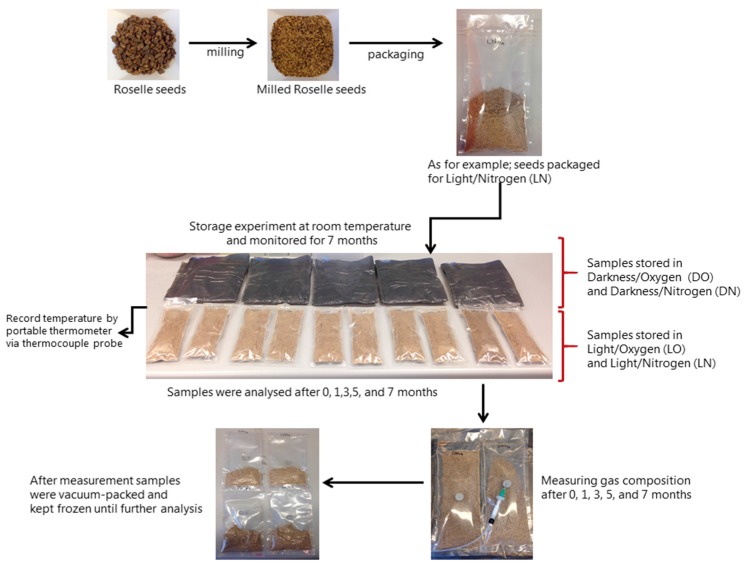
Diagram of packaging and storage conditions.

The samples stored in light were placed in a room without direct sunlight and with only electric light (Philips Master TL-D Super 80, 36 W/380, Copenhagen, Denmark) turned on constantly. The samples that were stored in darkness were kept in the same room inside two black plastic bags. The samples were stored at room temperature during the entire storage period. During the storage experiment, the temperature readings were recorded by a portable thermometer (EL-EnviroPad-TC, Corintech, UK) via a thermocouple probe. A diagram of packaging and storage conditions is shown in Figure 8. The minimum, average, and maximum of the storage temperatures were 20.3 °C, 22.2 °C, and 24.2 °C, respectively. The samples were analyzed after approximately 0, 1, 3, 5, and 7 months of storage (or exactly 0, 32, 101, 168, and 225 days, respectively). In the following, the time points will be given as the approximate number of months. The two replicates of each storage condition were analyzed on each day of analysis and, from each of these, two sub-replicates were prepared for all the analyses. Overall, this resulted in four replicates for each storage treatment on each day of analysis. In the sample preparation and the analysis steps, the handling of the samples was randomized. Four samples resulted in very atypical values and were excluded from the dataset.

### 3.5. Gas Composition

Before the bags were opened for analysis, the gas composition was measured using a CheckMate 9900 (PBI Dansensor A/S, Ringsted, Denmark). After measurement, the sample was vacuum-packed and kept frozen until further analysis. The overall gas composition during the entire storage period is shown in Figure 1. 

### 3.6. Volatile Compound Analysis

Dynamic headspace sampling (DHS) was applied to extract volatile compounds. The method was carried out as described by Juhari [19]. Twenty-five gram of milled seeds was put in a gas washing flask (500 mL) with a magnetic stirrer. The samples were equilibrated to 40 °C ± 1 °C in a circulating water bath and then purged with nitrogen (100 mL min^−1^) for 40 min. Volatile compounds were collected on a Tenax-TA traps (Markes International, Llantrisant, UK).

The trapped volatiles were thermally desorbed (TurboMatrix 350, Perkin Elmer, Shelton, CT, USA) and transferred to a gas chromatography-mass spectrometry (7890A GC-system interfaced with a 5975C VL MSD from Agilent Technologies, Palo Alto, CA, USA) including a DB-Wax column (30 m × 0.25 mm × 0.50 µm). Peak areas and mass spectra were extracted from the chromatograms using the PARAFAC2 based software PARADISe (University of Copenhagen, Copenhagen, Denmark) [26] and mass spectra were identified using the NIST05 database. Peak areas were used as relative measures of concentration. Volatile compound identification was confirmed by comparison with retention indices (RI) of authentic reference compounds or retention indices reported in the literature.

### 3.7. Data Analysis

One-way analysis of variance (ANOVA) was performed using the software JMP (version 13.0, SAS Institute Inc., Cary, NC, USA). Principal component analysis (PCA) with autoscaling was run using the Latentix software (LatentiX 2.0. Latent5, Copenhagen, Denmark). 

## 4. Conclusions

To our knowledge, this is the first time that changes of volatile compounds during storage of milled Roselle seeds are reported.

The present study demonstrates that milled Roselle seeds are a potential ingredient for food purposes because they contain high levels of protein, lipid and total dietary fiber. Besides that, we have shown that milled Roselle seeds exhibit good water and oil absorption capacity, which can be beneficial if included in food products. Furthermore, this study points out the importance of controlling the storage conditions to conserve the quality of milled Roselle seeds. For optimal storage, milled Roselle seeds should be stored in darkness rather than light, and flushed with nitrogen because light and oxygen accelerate the oxidation processes in the packaged food. Storage under these conditions may provide a shelf life of three months without attaining off-flavor.

## Figures and Tables

**Table 1:** Proximate composition, water and oil absorption capacity of different Roselle seed types.

Type of Analysis	Current Study	Previous Studies								
	Malaysian	Egyptian ^1^	Egyptian ^2^			Indian ^3^		Malaysian ^4^		
	Sun Dried		Light Red	Early Dark Red	Late Dark Red	Amw-2	Bhimili-1	Raw Freeze Dried	Sun Dried	Boiled Sun Dried
Moisture content (%)	8.4 ± 0.1	7.6	9.3	11.7	11.5	8.6	6.7	6.8	9.9	9.8
Ash (%)	6.5 ± 0.0	7.0	6.9	5.8	6.5	5.4	5.4	7.4	7.5	6.6
Lipid (%)	16.2 ± 0.5	22.3	21.6	22.5	23.3	19.1	22.8	27.22	22.1	29.6
Protein (%)	21.3 ± 0.9	15.4	31.0	30.1	30.9	18.8	22.3	35.4	33.5	30.6
Total dietary fiber (%)	47.3 ± 1.4	ND	ND	ND	ND	42.6	39.5	25.5	18.3	19.2
Crude fiber (%)	ND	15.3	4.1	3.4	1.2	ND	ND	ND	ND	ND
Carbohydrate (%)	0.3 ± 0.1	ND	36.4	38.1	38.1	ND	ND	2.3	13.0	4.0
Water absorption capacity (%)	174.5 ± 2.8	ND	254	254	220	ND	ND	ND	ND	ND
Oil absorption capacity (%)	144.0 ± 4.2	ND	159	158	125	ND	ND	ND	ND	ND

^1^ Study done by Samy [17], ^2^ El-Adawy and Khalil [6], ^3^ Rao [2], and ^4^ Hainida [18]. ND indicates not determined.

**Table 2:** Volatile compounds identified by Gas Chromatography-Mass Spectrometry (GC-MS) in the packed milled Roselle (*Hibiscus sabdariffa* L.) seeds stored for seven months in the presence or absence of oxygen and under different light conditions (light or darkness). Volatiles in bold were detected in our previous work on aroma profile of Roselle seeds [19].

Compounds		Retention Index		Significance ^h^			Odor Description (from Literature)
		Exp	Auth/Litt ^a^	Storage	Oxygen	Light	
***Aldehydes***							
**1**	**2-Methylpropanal**	**811**	**770–834**	***	*	**ns**	**Pungent, burnt, malty, grain ^e^**
**2**	**Butanal**	**873**	**830–911**	***	**ns**	***	**Green, plastic-like ^c^, floral ^d^**
**3**	**2-Methylbutanal**	**911**	**Auth**	***	***	**ns**	**Cocoa, almond, malty ^b^, fermented ^e^**
**4**	**3-Methylbutanal**	**915**	**Auth**	***	***	**ns**	**Fruity, toasted, malty, green ^c^**
**5**	**Pentanal**	**979**	**Auth**	***	***	*	**Almond, malty, pungent ^c^, oily, green ^e^**
6	2-Methylpentanal	989		***	***	ns	Unpleasant, rotten apples ^c^
**7**	**Hexanal**	**1093**	**Auth**	***	***	**ns**	**Grassy, tallow, fatty ^b^**
8	2-Methyl-(*E*)-2-butenal	1092		***	***	ns	Green, pungent, nutty, ethereal ^d^
**9**	**Octanal**	**1303**	**Auth**	***	***	*	**Citrus, waxy ^d^**
10	(*E*)-2-Heptenal	1335	1273–1366	***	**	***	Green, vegetable, fatty ^d^
11	2-Ethyl-2-hexenal	1345	1322	***	***	ns	Fatty, citrus, green ^b^, grassy, soapy ^e^
12	Nonanal	1399	Auth	***	***	ns	Fatty ^e^
**13**	**(*E*)-2-Octenal**	**1437**	**1393–1467**	***	**	**ns**	**Green, nutty, fatty ^b^, waxy, mushroom ^c^**
**14**	**Benzaldehyde**	**1532**	**Auth**	***	**ns**	*	**Almond, burnt, sugar ^b^**
15	Benzeneacetaldehyde	1653	1592–1684	**	***	ns	Green, hyacinth ^d^
16	2-Butyl-2-octenal	1679	1640–1688	***	*	ns	Green, citrus, grassy, fruity ^g^^,^*
17	2,4-Nonadienal	1714	1660–1740	***	**	ns	Watermelon, fatty, waxy, green ^b^
18	2,4-Decadienal	1826	1763–1858	***	ns	ns	Fatty, fried ^d^
***Alcohols***							
**19**	**2-Propanol**	**935**	**884–975**	**ns**	**ns**	**ns**	**Alcoholic, musty, woody ^d^**
**20**	**2-Butanol**	**1036**	**989–1057**	**ns**	**ns**	***	**Fruity, sweet, apricot ^d^**
**21**	**Propanol**	**1049**	**Auth**	**	***	**ns**	**Alcoholic, fermented ^d^**
**22**	**2-Methyl-1-propanol**	**1105**	**Auth**	***	***	**ns**	**Ethereal, winey, whiskey ^d^**
23	1-Methoxy-2-propanol	1138	1108–1160	*	ns	ns	Mild, ethereal, weak pleasant ^f^
**24**	**Butanol**	**1162**	**Auth**	***	***	**ns**	**Fusel, oily ^d^**
25	1-Penten-3-ol	1175	1112–1207	***	***	ns	Pungent, buttery, milky ^c^
**26**	**3-Methyl-1-butanol**	**1222**	**Auth**	***	***	**ns**	**Fermented, fusel, alcoholic, whiskey ^d^**
**27**	**Pentanol**	**1271**	**Auth**	***	**	**ns**	**Alcohol, pungent, fruity, balsamic ^b^**
**28**	**2-Heptanol**	**1339**	**1280–1344**	***	**	*	**Citrus, herbal, floral, green ^d^**
**29**	**Hexanol**	**1369**	**Auth**	***	**ns**	*	**Resin, flowery, green ^b^**
30	2-Octanol	1431	1380–1430	***	***	*	Spicy, fresh, green, woody, earthy ^d^
31	1-Octen-3-ol	1460	Auth	***	*	***	Mushroom, earthy ^c^
**32**	**Octanol**	**1570**	**Auth**	**ns**	**ns**	**ns**	**Mushroom, moss, nutty, burnt ^b^**
33	2,3-Butanediol	1592	1492–1620	***	***	ns	Fruity, creamy, buttery ^d^
**34**	**2-Decanol**	**1628**	**1585–1622**	**	**	**ns**	**Fatty, waxy, floral, orange, sweet ^d^**
35	Benzyl alcohol	1892	Auth	***	ns	*	Sweet, flowery ^b^
36	Phenethyl alcohol	1929	Auth	ns	ns	ns	Honey, spice, rose, lilac ^b^
***Ketones***							
37	Acetone	814	775–847	***	*	ns	Solvent, ethereal ^d^
**38**	**2-Butanone**	**903**	**Auth**	***	*	***	**Chemical-like, ether-like, cheesy ^c^**
39	3-Methyl-2-butanone	925	918–989	***	***	ns	Acetone-like ^f^
40	1-Penten-3-one	1018	973–1056	***	***	ns	Pungent, rotten, fruity, plastic-like ^c^
**41**	**2-Octanone**	**1298**	**Auth**	***	***	**ns**	**Soap, gasoline-like ^b^**
**42**	**6-Methyl-5-hepten-2-one**	**1349**	**1296–1368**	***	***	**ns**	**Mushroom, woody, rubbery ^f^, fruity ^e^**
**43**	**2-Nonanone**	**1396**	**Auth**	***	***	**ns**	**Hot milk, soap, green, blue cheese ^b^**
44	3-Octen-2-one	1415	1392–1411	***	***	ns	Nutty, blueberry, oily, fruity, green ^e^
**45**	**2-Decanone**	**1502**	**1463–1519**	***	***	**ns**	**Floral, orange, fatty, peach ^d^**
46	3-Nonen-2-one	1522	1523	***	*	**	Fruity, berry, brandy, mushroom ^d^
47	3,5-Octadien-2-one	1581	1521–1610	*	ns	ns	Fruity, green, grassy ^d^
***Furans***							
48	2-Ethylfuran	948	923–975	***	**	ns	Rubbery, pungent, acid, sweet ^c^
49	2-Propylfuran	1030	1011–1043	***	ns	ns	Heated peanut, apricot, plum ^f^
50	2-Butylfuran	1143	1088–1140	***	ns	ns	Mild, fruity, wine, sweet, spice ^c^
**51**	**2-Pentylfuran**	**1242**	**1193–1258**	***	**ns**	**	**Green bean, buttery ^b^, green, pungent ^e^**
52	2-Hexylfuran	1343	1312–1345	***	ns	ns	-
53	2-Heptylfuran	1440	1416–1454	***	ns	ns	Green, fatty, oily, roasted nutty ^d^
54	Furfural	1472	Auth	*	ns	ns	Bread, almond, sweet ^b^, fruity, wood ^c^
55	Dihydro-4,5-dimethyl-2(3H)-furanone	1619	1590–1624	***	***	ns	Caramel, sweet, candy ^d^
***Acids***							
56	Acetic acid	1446	1401–1485	***	***	ns	Vinegar ^b^
57	Propanoic acid	1545	1487–1570	ns	ns	ns	Pungent, rancid, soy ^b^
58	2-Methylpropanoic acid	1574	1520–1608	***	***	ns	Acidic, sour, cheesy, dairy, rancid ^d^
59	Butanoic acid	1634	1556–1674	***	***	ns	Cheesy, sharp, acetic, buttery, fruity ^d^
60	2-Methylbutanoic acid	1678	1638–1706	***	ns	ns	Acidic, pungent, cheesy ^d^
61	3-Methylbutanoic acid	1676	1631–1707	***	*	ns	Cheesy, sour, sweaty, tropical ^d^
62	Pentanoic acid	1745	1686–1766	***	ns	ns	Cheesy, acidic, sweaty, rancid ^d^
63	Hexanoic acid	1854	1797–1880	***	*	ns	Sweaty, cheesy, rancid, goat-like ^c^
64	Heptanoic acid	1961	1913–2000	***	ns	ns	Cheesy, rancid, sour, sweaty ^d^
65	Octanoic acid	2071	2011–2100	*	ns	ns	Fatty, rancid, vegetable, cheesy ^d^
***Esters***							
**66**	**Ethyl acetate**	**891**	**850–914**	***	***	**ns**	**Pineapple ^b^, fruity, orange ^c^**
67	Ethyl pentanoate	1147	Auth	***	***	ns	Fruity, apple, pineapple, green ^d^
68	Ethyl lactate	1357	1342–1356	ns	ns	ns	Fruity, creamy ^d^
69	Methyl acetate	826	782–877	***	***	***	Fruity, solvent-like, blackcurrant ^c^
70	Methyl octanoate	1397	Auth	***	*	**	Orange ^b^
71	Methyl nonanoate	1500	1476–1536	***	**	***	Coconut ^b^
***Terpenes***							
72	α-Pinene	1014	Auth	***	***	*	Herbal, pine, woody ^d^
73	Eucalyptol	1207	1173–1246	***	**	*	Herbal, eucalyptus, minty
74	α-Terpineol	1711	Auth	***	***	ns	Pine, lilac, citrus, woody, floral ^d^
***Pyrazines***							
75	2,6-Dimethylpyrazine	1342	1300–1355	***	***	ns	Cocoa, roasted, nutty ^d^
76	Tetramethyl pyrazine	1490	1438–1486	***	ns	*	Nutty, musty, chocolate, coffee ^e^
***Sulfur-containing compounds***							
77	Dimethyl sulfide	743	715–804	ns	***	**	Sulfurous, onion, sweet corn ^d^
78	Dimethyl disulfide	1068	1036–1127	*	***	*	Sulfurous, cabbage, onion ^d^
***Lactones***							
**79**	**gamma-Butyrolactone**	**1642**	**1593–1673**	***	**ns**	*	**Creamy, oily, fatty ^d^**
80	gamma-Hexalactone	1718	1655–1745	***	ns	ns	Creamy, vanilla ^d^
81	gamma-Octalactone	1938	1867–1945	***	*	ns	Creamy, coconut ^d^
82	gamma-Nonalactone	2052	1981–2062	***	**	ns	Floral, waxy, metallic, plum ^d^
***Miscellaneous***							
**83**	**Phenol**	**2015**	**1949–2037**	**ns**	**ns**	**ns**	**Phenolic, medicinal ^c^**
84	Ethyl ether	618	570–619	***	ns	ns	Ethereal ^d^
85	Theaspirane	1555	1472–1553	ns	ns	ns	Herbal, tea, green, woody, spicy ^d^

^a^ Auth indicates that an authentic standard run on the same system had identical retention index (RI). Numbers are RI values reported in https://pubchem.ncbi.nlm.nih.gov for a polar capillary GC column; ^b^ Odor description based on Flavornet; ^c^ Odor description based on Pherobase; ^d^ Odor description based on The Good Scents Company; ^e^ Odor description based on Odor.org.uk; ^f^ Odor description based on PubChem; ^g^ Zhang et al. [23]; ^h^, *, **, *** indicate significance at *p* < 0.05, *p* < 0.01 and *p* < 0.001, respectively; ns means no significant difference between the samples.
